# Asymptotic Gaussian law for noninteracting indistinguishable particles in random networks

**DOI:** 10.1038/s41598-017-00044-8

**Published:** 2017-02-16

**Authors:** Valery S. Shchesnovich

**Affiliations:** Centro de Ciências Naturais e Humanas, Universidade Federal do ABC, Santo André, SP 09210-170 Brazil

## Abstract

For *N* indistinguishable bosons or fermions impinged on a *M*-port Haar-random unitary network the average probability to count *n*_1_, … *n*_*r*_ particles in a small number *r* ≪ *N* of binned-together output ports takes a Gaussian form as *N* ≫ 1. The discovered Gaussian asymptotic law is the well-known asymptotic law for distinguishable particles, governed by a multinomial distribution, modified by the quantum statistics with stronger effect for greater particle density *N*/*M*. Furthermore, it is shown that the same Gaussian law is the asymptotic form of the probability to count particles at the output bins of a fixed multiport with the averaging performed over all possible configurations of the particles in the input ports. In the limit *N* → ∞, the average counting probability for indistinguishable bosons, fermions, and distinguishable particles differs only at a non-vanishing particle density *N*/*M* and only for a singular binning *K*/*M* → 1, where *K* output ports belong to a single bin.

## Introduction

Indistinguishable identical particles show correlated behavior due to their quantum statistics even in the absence of interactions: indistinguishable bosons show bunching, e.g., leave the balanced beam splitter in the same port, as demonstrated in the famous experiment with single photons^1^ and recently also with massive bosons^2^, while indistinguishable fermions show anti-bunching^3^. Fermionic (bosonic) behavior can also be emulated with bosons (fermions) by using entangled particles^4, 5^. Scaling up from two indistinguishable particles on a beam splitter to many particles in large-size multiports significantly increases complexity of behavior, as shown theoretically^6–11^ and demonstrated in a series of spectacular experiments^11–16^. Calculating the probabilities or reproducing the statistics of output configurations of noninteracting indistinguishable bosons in a large optical multiport requires exponential resources in the number of particles, which is the essence of the Boson Sampling idea^17^ (and its generalizations^18, 19^), where with a few dozens of indistinguishable photons one could have the computational supremacy over the best of current digital computers^17^. The proof-of-principle experiments of several groups open a way to build such a device^20–27^.

The behavior of identical particles in multiports is an interplay between the quantum statistics and interference. On the one hand, indistinguishable particles in multi-port networks show correlated behavior beyond the quantum statistics, e.g., in the symmetric (Bell type) multiports complex multi-particle interference results in common forbidden output configurations both for bosons and fermions^10^, confirmed recently with photons^16^. On the other hand, the quantum statistics shows up as the two-particle correlations at the Anderson localization in propagation through a disordered media^28–30^, induces the “photon clouding” in a unitary optical multiport^24^, defines the moments of the output distribution in the scattering of identical particles in chaotic cavities^31^, and is responsible for the generalized boson bunching and fermion anti-bunching^32^.

A natural question arises: Is there a universal statistics-dependent law in behavior of noninteracting indistinguishable identical bosons (fermions) in multi-port networks? It is shown below that indeed the probability of counting indistinguishable particles in binned-together output ports of a unitary *M*-port, averaged over the Haar-random unitary matrix representing the multiport, has a statistics-dependent asymptotic Gaussian form as *N* ≫ 1, where the quantum statistics enters through the particle density *N*/*M*. (Our main interest is the bosonic case, since indistinguishable bosons and distinguishable particles can share the same port in a multiport, whereas we also consider fermions to identify the contribution of the quantum statistics). This quantum asymptotic law is reminiscent of the well-known asymptotic law for a multinomial distribution (the de Moivre-Lagrange-Laplace theorem^33^ below), which governs an analogous setup with distinguishable (or classical) particles. In the thermodynamic limit *N* → ∞, the average probabilities of counting indistinguishable bosons, fermions, and distinguishable particles differ only for a finite particle density *α* = *N*/*M* and a singular binning, i.e., *K*/*M* → 1 with *K* output ports belonging to a single bin.

## Results

### Asymptotic Gaussian law for distinguishable particles in a random network

**Figure 1:**
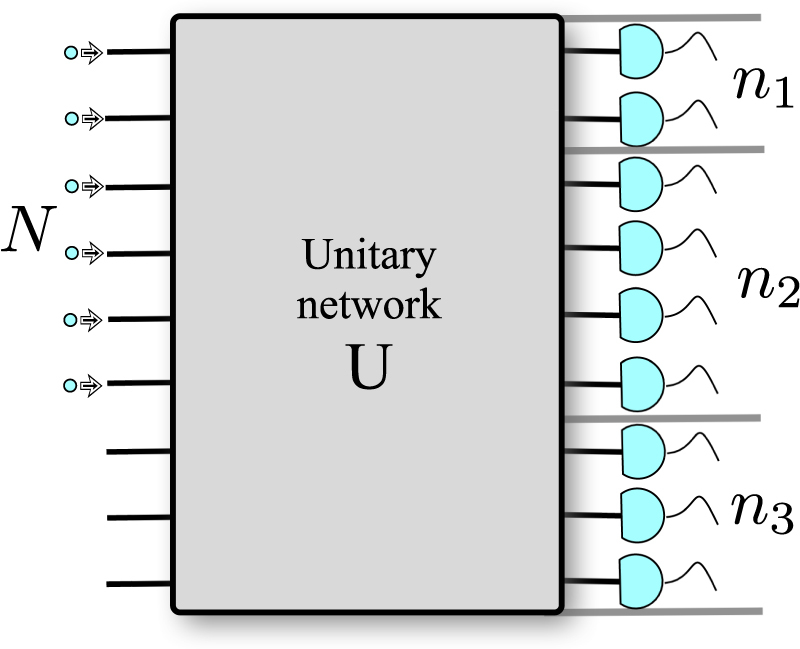
Unitary network with the output ports grouped into bins. A quantum network, having a unitary matrix *U*, with *N* indistinguishable identical particles at its input and binned-together output ports (three bins in this case). We are interested in the probability of counting **n** = (*n*_1_, *n*_2_, *n*_3_) particles in the output bins. (Two or more bosons as well as classical particles may share the same input port).

Consider a unitary *M*-port network *U* Fig. 1, whose output ports are partitioned into *r* bins having **K** ≡ (*K*_1_, …, *K*_*r*_) ports, with *N* noninteracting identical particles at the input. Let us begin with the case of distinguishable particles impinging on a multiport. Here by the term “distinguishable particles” we mean the identical particles in different states with respect to the degrees of freedom not affected by a multiport^1, 8, 34^, such as the arrival time in the case of photons (e.g., particles sent one at a time through the multiport). The probability for a single particle from input port *k* to land into bin *i* reads ${p}_{i}={\sum }_{l\in {K}_{i}}p(l|k)={\sum }_{l\in {K}_{i}}{|{U}_{k,l}|}^{2}$, where *p*(*l*|*k*) = |*U*_*k*,*l*_|^2^ is the probability of the transition *k* → *l*. Below we will be interested in the average probability in a random unitary multiport (except where stated otherwise, here and below the term “average” and the notation 〈…〉 means the average over the Haar-random unitary matrix *U*). A random unitary optical multiport can be experimentally realized with a very high fidelity^25^ and without explicit matrix calculations^35^. We have 〈*p*_*i*_〉 = *q*_*i*_ ≡ *K*_*i*_/*M*, since 〈|*U*_*kl*_|^2^〉 = 1/*M* (see Supplementary Information). For identical particles sent one at a time through a random multiport, the average probability to count **n** ≡ (*n*_1_, …, *n*_*r*_) particles in the output bins becomes a multinomial distribution

1 $$\langle {P}^{(D)}({\bf{n}})\rangle =\frac{N!}{\displaystyle \prod _{i=1}^{r}{n}_{i}!}\prod _{i=1}^{r}{q}_{i}^{{n}_{i}}\mathrm{.}$$

Eq. (1) turns out to be also a good approximation to the average counting probability for *N* distinguishable bosons or fermions at the input (as the numerical results presented below show), e.g., as in propagation of distinguishable particles in the disordered media^28–30^ or chaotic cavities^31^. However, Eq. (1) cannot give the exact average probability for these cases, since for *N* simultaneous distinguishable particles at the input there is an extra factor (see Supplementary Information) due to the correlations between the matrix elements |*U*_*kl*_|^2^.

The classic result in the probability theory, below referred to as the de Moivre-Lagrange-Laplace theorem^33^, states that given *q*_1_, …, *q*_*r*_, such that *q*_*i*_ ≠ 0 and ∑_*i*_ *q*_*i*_ = 1, the probability of Eq. (1) takes asymptotically the following multivariate Gaussian form as *N* → ∞ (more details in Methods)

2 $${{\cal{G}}}^{(D)}({\bf{n}})=\frac{\exp \{-\displaystyle \sum _{i=1}^{r}\frac{{({n}_{i}-N{q}_{i})}^{2}}{2N{q}_{i}}\}}{{(\mathrm{2}\pi N)}^{\frac{r-1}{2}}\displaystyle \prod _{i=1}^{r}\sqrt{{q}_{i}}}\mathrm{.}$$

In other words, for *N* ≫ 1 and *r* ≪ *N*, the numbers *n*_1_, …, *n*_*r*_ decompose as follows ${n}_{i}^{(D)}={q}_{i}N+\sqrt{N}{y}_{i}$, where **y** = (*y*_1_, …, *y*_*r*_) is a vector of random variables, constrained by $\displaystyle {\sum }_{i=1}^{r}{y}_{i}=0$, with the joint probability density *ρ*(**y**) of a Gaussian form^36^ (see also Methods):

3 $$\rho ({\bf{y}})=\frac{\exp \{-\displaystyle \sum _{i=1}^{r}\frac{\displaystyle {y}_{i}^{2}}{2{q}_{i}}\}}{{(\mathrm{2}\pi )}^{\frac{r-1}{2}}\displaystyle \prod _{i=1}^{r}\sqrt{{q}_{i}}},\quad \langle {y}_{i}\rangle =\mathrm{0,}\quad \langle {y}_{i}{y}_{j}\rangle =\sqrt{{q}_{i}{q}_{j}}{\delta }_{ij}-{q}_{i}{q}_{j}\mathrm{.}$$

One important note. For a fixed unitary multiport *U*, averaging the transition probability over the input port gives ${\langle p(l|k;U)\rangle }_{k}\equiv \mathrm{1/}M\displaystyle {\sum }_{k=1}^{M}{|{U}_{kl}|}^{2}=\mathrm{1/}M={\langle p(l|k;U)\rangle }_{U}$ (the averaging over the input port *k* and over the Haar-random unitary matrix *U* give the same result). Hence Eqs (1) and (2) apply also for any unitary multiport with the averaging performed over all possible configurations of distinguishable particles in the input ports (i.e., over **k** = (*k*_1_, …, *k*_*N*_), 1 ≤ *k*_*i*_ ≤ *M*).

An intriguing question is: What is the quantum version of the above asymptotic result, i.e., what form the analogous asymptotic average probability has if *N indistinguishable* identical particles are at the input? Since the averaging over the random multiport matrix is performed, one would expect that the multiport-specific quantum interference effects do not show up in the result, whereas the difference in counting the classical (distinguishable) and quantum (indistinguishable) particles in the output bins is solely due to the quantum statistics. It turns out that in the quantum case the asymptotic form of the counting probability is the Gaussian law of Eq. (2) modified by the quantum statistics through the particle density *α* ≡ *N*/*M*.

### Asymptotic Gaussian law for indistinguishable particles in a random network

Let us consider bosons and fermions simultaneously (for fermions only up to one particle can be found per network port, as in Fig. 1). For indistinguishable bosons (fermions), the unitary invariance of the Haar measure makes the average probability uniform over the input/output configurations (see Supplementary Information), i.e., for an input **k** = (*k*_1_, …, *k*_*N*_) and an output **l** = (*l*_1_, …, *l*_*N*_) configurations the average probability of the transition **k** → **l** is just the inverse of the number of Fock states of *N* bosons (fermions) in *M* ports: $\langle {p}^{(B)}({\bf{l}}|{\bf{k}})\rangle =\frac{N!}{(M+N-\mathrm{1})\ldots M}$ $(\langle {p}^{(F)}({\bf{l}}|{\bf{k}})\rangle =\frac{N!}{M\ldots (M-N+\mathrm{1})})$. Summing up the number of the transitions corresponding to a given counting result **n** of *N* bosons (fermions) in *r* bins, we get the following quantum equivalent of Eq. (1) (with the upper signs for bosons and the lower ones for fermions)

4 $$\begin{array}{rcl}\langle {P}^{(B,F)}({\bf{n}})\rangle  & = & \frac{N!}{(M\pm N\mp \mathrm{1})\ldots M}\prod _{i=1}^{r}\frac{({K}_{i}\pm {n}_{i}\mp \mathrm{1})\ldots {K}_{i}}{{n}_{i}!}\\  & = & \langle {P}^{(D)}({\bf{n}})\rangle \frac{\displaystyle \prod _{i=1}^{r}\displaystyle \prod _{l=1}^{{n}_{i}-1}(1\pm l/{K}_{i})}{\displaystyle \prod _{l=0}^{N-1}(1\pm l/M)},\end{array}$$

where the classical 〈*P* ^(*D*)^(**n**)〉 and quantum factors are separated. It turns out that the quantum factor in Eq. (4) can be approximated as *N* → ∞ in the same way as the classical probability in Eq. (1), i.e., there is also an asymptotic Gaussian law for Eq. (4). Namely, for any partition of the output ports into *r* bins, the probability 〈*P* ^(*B*,*F*)^(**n**)〉 has the following asymptotic form as *N* → ∞ (see Methods for the derivation)

5 $${{\cal{G}}}^{(B,F)}({\bf{n}})=\frac{\exp \{-\displaystyle \sum _{i=1}^{r}\frac{{({n}_{i}-N{q}_{i})}^{2}}{2[1\pm \alpha ]N{q}_{i}}\}}{{(2\pi [1\pm \alpha ]N)}^{\frac{r-1}{2}}\displaystyle \prod _{i=1}^{r}\sqrt{{q}_{i}}}$$

with *α* = *N*/*M*. For the Gaussian law (5) to be a good approximation to the probability (4) for *N* ≫ 1 the number of bins must satisfy *r* ≪ *N*, min(*K*_*i*_) (see Methods).

It follows from Eq. (5) that for *N* ≫ 1 the *i*th bin occupation number *n*_*i*_ admits the following decomposition ${n}_{i}^{(B,F)}={q}_{i}N+\sqrt{[1\pm \alpha ]N}{y}_{i}$, where (*y*_1_, …, *y*_*r*_) *is the same* set of Gaussian random variables as in Eq. (3). Therefore, as *N* → ∞ we get *x*_*i*_ ≡ *n*_*i*_/*N* → *q*_*i*_, *i* = 1, …, *r* for indistinguishable bosons (for a finite density *α* < ∞), fermions, and distinguishable particles. For bosons there is also the asymptotically infinite density case, *N* → ∞ for a fixed *M ≫ 1*, resulting in a qualitatively different (non-deterministic) limit ${x}_{i}\to {q}_{i}+{y}_{i}/\sqrt{M}$. It is known^37^ that in this case the output probabilities *p* ^(*D*)^(**l**|**k**; *U*) and *p* ^(*B*)^(**l**|**k**; *U*) of the transition **k** → **l** for distinguishable particles and indistinguishable bosons, respectively, differ even asymptotically as *N* → ∞ (except for the trivial cases, such as *U* being a permutation matrix).

The asymptotic Gaussian laws (2) and (5) apply for an arbitrary assignment of output ports to bins, while keeping the total number of ports in each bin fixed. Since the average probability 〈*P* ^(*B*,*F*)^(**n**)〉 (4) depends only on the number of Fock states corresponding to the particle counts **n**, the asymptotic Gaussian law (5) is valid also for an arbitrary assignment of *output configurations* **l** = (*l*_1_, …, *l*_*N*_), allowed by statistics, to $\frac{(N+r-1)!}{(r-1)!N!}$ *bins of configurations* enumerated by a vector index **n** = (*n*_1_, …, *n*_*r*_) (replacing the vector of particle counts in the bins of the output ports), if the total number of configurations **l** in bin **n** converges as *N* → ∞ to the number of configurations corresponding to counting **n** particles in *r* bins of the output ports.

Similarly as in the classical case, the probability formula (4) and the asymptotic Gaussian law (5) apply also to *any given* unitary multiport with the averaging performed over the input configurations allowed by the quantum statistics. Indeed, the quantum transition probability, **k** → **l**, satisfies the time-inversion relation *p* ^(*B*,*F*)^(**l**|**k**; *U*) = *p* ^(*B*,*F*)^(**k**|**l**; *U* ^†^), thus averaging it over the allowed input configurations **k** produces a result uniform in **l** and equal to that obtained by averaging over the Haar-random unitary *U* (see Methods).

### Comparison of the asymptotic Gaussian laws with the corresponding probabilities

To compare the asymptotic Gaussian law with the corresponding probability of particle counting let us first give the formula for the respective probability *P*(**n**; *U*) in an arbitrary multiport *U*. Suppose for the moment that the particles are distinguishable (one can trace the path of each particle through a network by the distinct values of some internal degree(s) of freedom of the particles). Introduce for each output bin a *N*-dimensional positive semi-definite Hermitian matrix ${H}_{a,b}^{(i)}\equiv {\sum }_{l\in {K}_{i}}{U}_{{k}_{a},l}{U}_{{k}_{b},l}^{\ast }$, with **k** = (*k*_1_, …, *k*_*N*_) being the vector of the input ports of the particles, and assume that **a**_1_, …, **a**_*r*_ is a partition of (1, …, *N*) into *r* subsets, such that vector **a**_*i*_ contains *n*_*i*_ indices identifying the particles which end up in the *i*th bin. Then the probability of counting **n** = (*n*_1_, …, *n*_*r*_) particles in the output bins becomes

6 $${P}^{(D)}({\bf{n}};U)=\sum _{{{\bf{a}}}_{1},\ldots ,{{\bf{a}}}_{r}}\prod _{i=1}^{r}\prod _{a\in {{\bf{a}}}_{i}}\displaystyle {H}_{a,a}^{(i)}\mathrm{.}$$

In the quantum case, the fact that it is impossible to trace the individual paths of indistinguishable particles through a network results in the cross-particle interference terms, weighted by a function *J*(*σ*) acting on the group ${{\cal{S}}}_{N}$ of permutations *σ* of *N* objects^32, 34^ (see Supplementary Information): *J* ^(*B*)^(*σ*) = 1 for indistinguishable bosons and *J* ^(*F*)^(*σ*) = sgn(*σ*) for fermions. For an input configuration **k** with *s*_*j*_ particles (0 ≤ *s*_*j*_ ≤ *N* for bosons and 0 ≤ *s*_*j*_ ≤ 1 for fermions) in input port *j*, *j* = 1, …, *M*, we obtain (see Methods)

7 $$P({\bf{n}};U)=\frac{1}{\displaystyle \prod _{j=1}^{M}{s}_{j}!}\sum _{\sigma \in {{\cal{S}}}_{N}}J(\sigma )\sum _{{{\bf{a}}}_{1},\ldots ,{{\bf{a}}}_{r}}\prod _{i=1}^{r}\prod _{a\in {{\bf{a}}}_{i}}\displaystyle {H}_{a,\sigma (a)}^{(i)}\mathrm{.}$$

Let us first test the convergence of the average probability 〈*P*(**n**)〉 to the corresponding asymptotic Gaussian law as *N* → ∞. Taking the three bin partition, see Fig. 1, as an illustrative example we numerically compare Eq. (1) to Eq. (2) and Eq. (4) to Eq. (5) (separately for bosons and fermions) by computing the maximum of the absolute value of the difference $|{\cal{G}}({\bf{n}})-\langle P({\bf{n}})\rangle |$ as a function of **n** = (*n*_1_, *n*_2_, *N* − *n*_1_ − *n*_2_) divided by the maximum value of 〈*P*(**n**)〉 (both the maximum of the Gaussian approximation ${\cal{G}}({\bf{n}})$ in Eqs (2) and (5) and the maximum of the average probability 〈*P*(**n**)〉 in Eqs (1) and (4) decrease with *N* for fixed *α* and *q*_1_, …, *q*_*r*_). The results are presented in Table 1, where we start with *N* = 12 and *M* = 36 (i.e., *α* = 1/3), increase *N* by factor 2 and compute the respective *M* and the partition (*K*_1_, *K*_2_, *K*_3_) for fixed *q*_1_ = *q*_3_ = 1/4 and *q*_2_ = 1/2 (these values are chosen to have integer partitions for all *M*).

**Table 1:** The numerically computed $\frac{{\rm{\max }}|{\cal{G}}({\bf{n}})-\langle P({\bf{n}})\rangle |}{{\rm{\max }}\langle P({\bf{n}})\rangle }$ for the distinguishable particles, bosons, and fermions for the three bin partition with *q*_1_ = *q*_3_ = 1/4 and *q*_2_ = 1/2.

*M*	*K*_1_	*K*_2_	*N*	$\frac{{\bf{\max }}|{{\bf{G}}}^{({\boldsymbol{D}})}({\bf{n}})-\langle {{\boldsymbol{P}}}^{({\boldsymbol{D}})}({\bf{n}})\rangle |}{{\bf{\max }}\langle {{\boldsymbol{P}}}^{({\boldsymbol{D}})}({\bf{n}})\rangle }$	$\frac{{\bf{\max }}|{{\bf{G}}}^{({\boldsymbol{B}})}({\bf{n}})-\langle {{\boldsymbol{P}}}^{({\boldsymbol{B}})}({\bf{n}})\rangle |}{{\bf{\max }}\langle {{\boldsymbol{P}}}^{({\boldsymbol{B}})}({\bf{n}})\rangle }$	$\frac{{\bf{\max }}|{{\bf{G}}}^{({\boldsymbol{F}})}({\bf{n}})-\langle {{\boldsymbol{P}}}^{({\boldsymbol{F}})}({\bf{n}})\rangle |}{{\bf{\max }}\langle {{\boldsymbol{P}}}^{({\boldsymbol{F}})}({\bf{n}})\rangle }$
36	9	18	12	0.096443	0.148050	0.075394
72	18	36	24	0.065787	0.102118	0.037101
144	36	72	48	0.045826	0.068456	0.025218
288	72	144	96	0.031497	0.047765	0.015706
576	144	288	192	0.022142	0.032836	0.010365
1152	288	576	384	0.015580	0.023029	0.006918

The maximum is taken over all distributions **n** = (*n*_1_, *n*_2_, *n*_3_) of *N* particles over the output bins.

**Figure 2:**
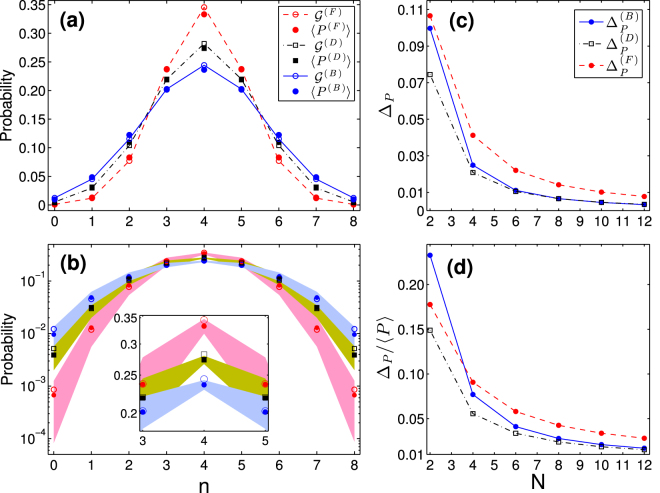
The Gaussian laws vs. the probabilities of particle counting in two output bins. Panel (a) shows the average probability (4) (filled circles) vs. the Gaussian law (5) (open circles), where bosons (fermions) correspond to the solid (dashed) line, and the classical average probability (1) (filled squares) vs. the Gaussian law (2) (open squares). Panel (b) shows the average probabilities (filled markers) and the Gaussian laws (open markers) together with the standard deviations; for a fixed *n*, the height of each filled region is twice the respective Δ_*P*_ of Eq. (8). Panel (c) shows the standard deviation Δ_*P*_ (8) at *n* = *N*/2 for bosons (the solid line), fermions (the dashed line), and distinguishable particles (the dash-dot line). Panel (d) shows the relative standard deviation Δ_*P*_/〈*P*〉 with 〈*P*〉 ≡ 〈*P*(*N*/2, *N*/2)〉. Here *q*_1,2_ = 1/2 and *α* = 1/3. In panels (a) and (b) *N* = 8, *M* = 24, and 0 ≤ *n* ≤ 8 corresponds to one of the bins.

To see how well the average probabilities (1) and (4) and the asymptotic Gaussian laws (2) and (5) compare with the corresponding particle counting probabilities for a randomly selected multiport matrix *U*, we numerically estimate the standard deviation employing the following formula

8 $$\displaystyle {{\rm{\Delta }}}_{P}^{2}=\frac{1}{{\cal{T}}}\sum _{j=1}^{{\cal{T}}}{[P({\bf{n}};{U}^{(j)})-\langle P({\bf{n}})\rangle ]}^{2}$$

with a large number ${\cal{T}}$ of randomly selected networks (the error controlled by comparing the results for independent data sets). Figure 2(a,b) gives the comparison of the Gaussian laws with the corresponding average counting probabilities of *N* = 8 particles in two equal-size bins *q*_1_ = *q*_2_ = 1/2 of the Haar-random multiports with *M* = 24. The filled regions in Fig. 2(b) give the standard deviation Δ_*P*_ numerically estimated using Eq. (8). Both the standard deviation Δ_*P*_ and also its relative value Δ_*P*_/〈*P*(**n**)〉 decrease with *N* at the peak *n*_*i*_ = *q*_*i*_ *N* of the Gaussian law (5), see Fig. 2(c,d), moreover, the standard deviation for bosons rapidly settles on that of distinguishable particles with increase of *N*.

#### Symmetries in the multiport matrix and a large deviation from the average probability

A multiport matrix *U* with symmetries may result in the counting probability *P*(**n**; *U*) having a large deviation (as compared with the standard deviation) from the respective average probability for particular input configurations. Let us consider as an example the Fourier multiport ${{\cal{U}}}_{kl}=\exp \{\frac{2i\pi }{M}kl\}\sqrt{M}$ ^6, 38^. In this case most of the output configurations **l** are forbidden for a cyclicly symmetric input configuration **k** ^10, 16^. For instance, with the help of numerical simulations it is found that for *M* = *N* ^2^ and an input satisfying the cyclic symmetry *k*_*j*+1_ = *k*_*j*_ + *N* (mod *M*), the equipartition of output ports into two bins (*q* = 1/2) produces the actual quantum distribution satisfying ${P}^{(B,F)}({\bf{n}};{\cal{U}})=\langle {P}^{(D)}({\bf{n}})\rangle$, i.e., equal to that of Eq. (1). Nevertheless, averaging over all allowed input configurations reproduces 〈*P* ^(*B*,*F*)^(**n**)〉, as it should be. In general, there are unitary matrices having symmetries and possessing many continuous parameters, e.g., the block-Hadamard unitary

9 $$U=\frac{1}{\sqrt{2}}\left(\begin{array}{cc}V & -V\\ V & V\end{array}\right),\quad V{V}^{\dagger }=I,$$

where *V* is an arbitrary unitary matrix. The unitary in Eq. (9) has forbidden laws for any *V*. For instance, for *N* = *M*/2 particles at the input and the equipartition of the output ports into two bins by the order of their indices, the *N*-dimensional Hermitian matrices which define the counting probability (7) are drawn from the following 2*N*-dimensional Hadamard ones

10 $${H}^{\mathrm{(1,2)}}=\frac{1}{2}\left(\begin{array}{cc}I & \pm I\\ \pm I & I\end{array}\right),$$

depending on the indices of the occupied input ports. As the result, some of the bin configurations (*n*_1_, *n*_2_) are forbidden for certain input configurations (e.g., for an even number of particles *N*, only even number of bosons *n*_1,2_ can be detected in either bin, whereas fermions are always divided into two halves *n*_1_ = *n*_2_ = *N*/2).

The Haar measure for the *M*-port unitary matrices has *M* ^2^ independent real parameters, where 2*M* − 1 of them are due to the invariance by multiplication with a diagonal unitary matrix^39^. The example in Eq. (10) shows that there exist continuous subsets of the unitary matrices which result in large deviations of the counting probability *P*(**n**; *U*) from the respective average. In general, for a fixed *U* we have in total (using Eqs (19) and (33) from Methods)

11 $${{\cal{V}}}_{total}=\frac{(N+r-1)!}{(r-1)!N!}=\frac{{N}^{r-1}}{(r-1)!}\prod _{i=1}^{r-1}(1+\frac{i}{N})=\frac{{N}^{r-1}{e}^{r-1}}{(r-1)!}[1+O(\frac{{r}^{2}}{N})],$$

values of *P*(**n**; *U*), whereas at most ${{\cal{V}}}_{ind}={(M-1)}^{2}={N}^{2}{(1/\alpha -1)}^{2}$ of the values could be independent (for fermions, when *K*_*i*_ ≥ *N* is not satisfied for all *i* = 1, …, *r*, some of the values of *P* ^(*F*)^(**n**; *U*) are zero by the Pauli exclusion principle). We get the upper bound ${{\cal{V}}}_{ind}/{{\cal{V}}}_{total}=O({N}^{3-r})$ as *N* → ∞, with *r* and *α* being fixed. Hence, the relative dimension of the subset of the unitary matrices with prescribed independent values of *P*(**n**; *U*) decreases with *N* at least for *r* ≥ 4 bins.

### Quantum to classical transition in the thermodynamic limit *N* → ∞

Quantum Statistical Mechanics predicts that, in the thermodynamic limit, for a system of weakly interacting identical particles the classical behavior appears at the vanishing density^40^ (*α* → 0 in our notations). Is there an analog of this rule for noninteracting indistinguishable particles in a random multiport? Let us call the boundary scaling *M* vs. *N* the largest scaling leading to the average probabilities in Eqs (1) and (4) being different in the limit *N* → ∞. Since the average transition probability 〈*p*(**l**|**k**)〉 decreases exponentially with *N* ^17^ (see Supplementary Information), to have a finite average probability 〈*P*(**n**)〉 one has to sum an exponential in *N* number of such transition probabilities. This observation together the fact that Eq. (5) applies to an arbitrary assignment of output configurations to the bins of configurations seem to lead to the conclusion that as *N* → ∞ the difference between the quantum (4) and classical (1) average probabilities vanishes (for a bounded particle density *α*). However, Eq. (5) and the above conclusion do not hold for a *singular binning*, the simplest of such is obtained by assigning *K* < *M* ports to bin 1 and the rest to bin 2 (or any number of bins), with *K*/*M* → 1 as *M* → ∞ (since *q*_*i*_ ≠ 1 is the applicability condition). In this case, setting ${H}_{i,j}={\sum }_{l=1}^{K}{U}_{{k}_{i},l}{U}_{{k}_{j},l}^{\ast }$ for the occupied input ports *k*_1_, …, *k*_*N*_, we obtain from Eq. (7) the probability to detect all *N* input particles in *K* output ports as follows^32^

12 $${P}^{(B)}={\rm{per}}(H),\quad {P}^{(F)}={\rm{\det }}(H),\quad {P}^{(D)}=\prod _{i=1}^{N}{H}_{i,i},$$

where per(…) stands for the matrix permanent^41^. The probability in Eq. (12) satisfies^32^ *P* ^(*F*)^ ≤ *P* ^(*D*)^ ≤ *P* ^(*B*)^ for any multiport and any *K* < *M* (this order of the probabilities is reflected also in Fig. 2(a,b) at the end-points *n* = 0 and *n* = *N*). The average values of the probabilities in Eq. (12) were reported before^32^. We have (we use Eq. (1) for distinguishable particles, which is a good approximation, Fig. 2)

13 $$\langle {P}^{(B,F)}\rangle ={(\frac{K}{M})}^{N}\prod _{l=1}^{N-1}(\frac{1\pm l/K}{1\pm l/M}),\quad \langle {P}^{(D)}\rangle ={(\frac{K}{M})}^{N},$$

where the upper (lower) sign stands for bosons (fermions). Let us assign *K* output ports to a single bin, such that *m* ≡ *M* − *K* is fixed as *K*, *M* → ∞ and assume that the input configuration contains up to one particle per input port. We get in the limit *N* → ∞ for *N*/*M* → *α* ≠ 0 (see Methods):

14 $$\langle {P}^{(B,F)}\rangle \mathop{\to }\limits_{N\to \infty }{(1\pm \alpha )}^{\mp m},\quad \langle {P}^{(D)}\rangle \mathop{\to }\limits_{N\to \infty }{e}^{-m\alpha }.$$

**Figure 3:**
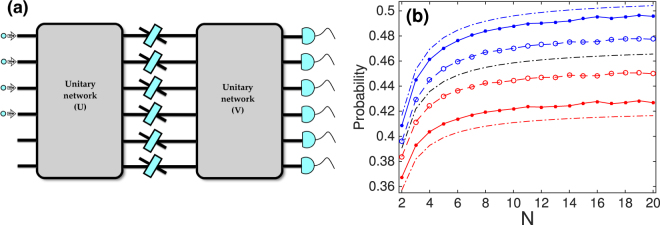
The survival probability in a lossy network. Panel (a): an equivalent representation of a lossy *M*-port linear network *A* = *UDV* with a unitary 2*M*-port network, where *U* and *V* are some unitary *M*-ports and the diagonal matrix $D={\rm{diag}}(\sqrt{{\eta }_{1}},\ldots ,\sqrt{{\eta }_{M}})$ corresponds to *M* beamsplitters (with the transmission coefficients *η*_1_, …, *η*_*M*_) placed between them. Panel (b): the average survival probability of *N* indistinguishable bosons (above the middle dash-dot line) and fermions (below the middle dash-dot line) in a random 4*N*-port network with *m* = 3 lossy channels having the loss rates *η*_1,2,3_ = 0.1 (dots on the solid lines) and *η*_1,2,3_ = 0.3 (open circles on the dashed lines). The dash-dot lines give the analytical average survival probability of Eq. (13) for *m* = 3 completely lossy channels *η*_1,2,3_ = 0, where from top the to bottom we have bosons, distinguishable particles, and fermions, respectively. The averages are computed over 9000 random unitary matrices *U*.

The result of Eq. (14) implies different survival probability of non-interacting identical particles in some lossy networks: even as *N* → ∞ the probability to detect *N* indistinguishable bosons (fermions) and distinguishable particles at the output of a “crowded” *M*-port network with *M* = *O*(*N*) can be different. Indeed, an arbitrary lossy linear network *A* (*AA* ^†^ ≠ *I*) can be equivalently represented by three consecutive multiports according to the singular-value decomposition *A* = *UDV* ^42^, where *U* and *V* are two unitary multiports, whereas $D={\rm{diag}}(\sqrt{{\eta }_{1}},\ldots ,\sqrt{{\eta }_{M}})$ corresponds to *M* beam splitters having the transmission coefficients *η*_1_, …, *η*_*M*_ connecting the multiports *U* and *V*, see Fig. 3(a). The commutation rules for the creation and annihilation operators require that 0 ≤ *η*_*k*_ ≤ 1 for a lossy multiport (see the Supplement to ref. 32). For a network with *m* strongly lossy channels *η*_1_ = … = *η*_*m*_ = 0 and *K* = *M* − *m* transparent ones (*η*_*m*+1_ = … = *η*_*M*_ = 1), by averaging over the Haar-random *U*, i.e., the path-dependence of the loss of particles (whereas *V* has no effect on the survival probability) we arrive at the survival probability given by Eq. (13) with the asymptotic results in Eq. (14). The conclusion extends to the networks having some of the loss rates satisfying *η* ≪ 1, while the others 1 − *η* ≪ 1, Fig. 3(b).

## Discussion

The existence of forbidden events common for bosons and fermions in the symmetric Bell multiports^10^ could be viewed as a manifestation of the primary role of the quantum interference over the quantum statistics. Now this view is complemented by the existence of the asymptotic statistics-dependent Gaussian law for indistinguishable particles in multiports for the counting probability in the binned-together output ports averaged either over the Haar-random multiports, or, for a fixed multiport, over the allowed input configurations of the particles (a quantum analog of the de Moivre-Lagrange-Laplace theorem^33^ for the multinomial distribution describing classical particles at the input). The asymptotic Gaussian law can have direct applications for noninteracting indistinguishable bosons or fermions in the setups where randomness plays a key role, such as the multiphoton propagation through disordered media^28–30^ and the multi-particle scattering in chaotic cavities^31^.

The rapid advance of the quantum technology has scaled up the number of identical particles and the size of employed networks in current experiments^11–16^, where a wealth of complex behavior is reported. A lot of attention is devoted to indistinguishable photons in multiports^19–27^ due to the Boson Sampling idea^17^ as a near-future feasible “sampling computer”, an alternative to the universal quantum computer^43^ for the demonstration of the quantum supremacy over the digital computers. Recently it was suggested^44^ that there might be also some decision problems easily solvable on a Boson Sampling device but not on the digital computers, where binning of the output configurations in a random multiport was proposed for devising such classically hard decision problems. While the complexity of behavior in the linear bosonic networks asymptotically challenges the digital computers, the search for simple asymptotic laws, such as the asymptotic Gaussian law of the present work, brings the dividends of understanding such a complex behavior.

## Methods

### Derivation of the quantum asymptotic Gaussian law

Derivation of the de Moivre-Lagrange-Laplace theorem can be based on the Stirling’s formula $n!=\sqrt{2\pi (n+{\theta }_{n})}{(n/e)}^{n}$, where $\frac{1}{6}< {\theta }_{n}< 1.77$ for *n* ≥ 1^45^ and *θ*_0_ = 1/(2*π*). It allows one to approximate the multinomial distribution of Eq. (1) as follows (here for *n*_*i*_ ≠ 0)

15 $$\frac{N!}{\displaystyle \prod _{i=1}^{r}{n}_{i}!}\prod _{i=1}^{r}\displaystyle {q}_{i}^{{n}_{i}}=\frac{\exp \{-N\displaystyle \sum _{i=1}^{r}{x}_{i}\mathrm{ln}(\frac{{x}_{i}}{{q}_{i}})\}}{{(2\pi N)}^{\frac{r-1}{2}}\displaystyle \prod _{i=1}^{r}\sqrt{{x}_{i}}}[1+O(\frac{r}{N})]$$

with *x*_*i*_ ≡ *n*_*i*_/*N*. Employing a meticulous error estimate^36^ as *N* → ∞ one can then derive the asymptotic result of Eq. (2). The main steps are the following. Observe that the Kullback-Leibler divergence ${D}_{KL}({\bf{x}}\parallel {\bf{q}})\equiv \displaystyle {\sum }_{i=1}^{r}{x}_{i}\mathrm{ln}({x}_{i}/{q}_{i})\ge 0$ in the exponent in Eq. (15) has a simple expansion for *x*_*i*_ close to *q*_*i*_ (its mean value)

16 $${D}_{KL}({\bf{x}}\parallel {\bf{q}})=\sum _{i=1}^{r}\{\frac{{({x}_{i}-{q}_{i})}^{2}}{2{q}_{i}}+O({|{x}_{i}-{q}_{i}|}^{3})\}\mathrm{.}$$

For *N* ≫ 1 and for small |*x*_*i*_ − *q*_*i*_| the first term on the right-hand side in Eq. (16) is a sufficient approximation for *D*_*KL*_(**x**||**q**) when the former is multiplied by a large negative factor (−*N*) and placed in the exponent in Eq. (15). In its turn, the factor *x*_*i*_ in the denominator on the right-hand-side of Eq. (15) can be replaced by *q*_*i*_ within the same approximation. The result is the stated Gaussian law

17 $$\frac{N!}{\displaystyle \prod _{i=1}^{r}{n}_{i}!}\prod _{i=1}^{r}\displaystyle {q}_{i}^{{n}_{i}}\approx \frac{\exp \{-N\displaystyle \sum _{i=1}^{r}\frac{{({x}_{i}-{q}_{i})}^{2}}{2{q}_{i}}\}}{{(2\pi N)}^{\frac{r-1}{2}}\displaystyle \prod _{i=1}^{r}\sqrt{{q}_{i}}}.$$

The above steps, besides clarifying a basic idea behind the proof of the classical asymptotic result, allow one to justify the analogous quantum asymptotic result by application of exactly the same approximations to the quantum average probability (4). Indeed, in the quantum case, additionally to the classical multinomial distribution of Eq. (15), there is the quantum factor

18 $${Q}^{(B,F)}\equiv \frac{\displaystyle \prod _{i=1}^{r}\displaystyle \prod _{l=0}^{{n}_{i}-1}(1\pm l/{K}_{i})}{\displaystyle \prod _{l=0}^{N-1}(1\pm l/M)}.$$

To estimate the latter, let us use the following result

19 $$\prod _{l=0}^{n}[1\pm \frac{l}{m}]={(1\pm \frac{n}{m})}^{n\pm m+1/2}{e}^{-n}[1+O(\frac{n}{(m\pm n)m})],$$

valid for all *n* ≥ 0 for the positive sign and for 0 ≤ *n* < *m* for the negative sign. Eq. (19) follows from the second-order Euler-Maclaurin summation formula^46^

20 $$\begin{array}{rcl}\sum _{l=1}^{n}f(l) & = & {\int }_{1}^{n}dxf(x)+\frac{f(n)+f(1)}{2}+\frac{f^{\prime} (n)-f^{\prime} (1)}{12}-\frac{1}{2}{\int }_{1}^{n}dx{B}_{2}(x)f^{\prime\prime} (x),\\ {B}_{2}(x) & \equiv  & 2{\int }_{0}^{x}dt{B}_{1}(t)+\frac{1}{6},\quad {B}_{1}(x)\equiv \{x\}-\frac{1}{2},\end{array}$$

where $-\frac{1}{12}\le {B}_{2}(x)\le \frac{1}{6}$ and {*x*} is the fractional part of *x*. Setting *f*(*x*) = ln(1 ± *x*/*m*) and using Eq. (20) one can derive Eq. (19). With the use of Eq. (19) the quantum factor is approximated uniformly over **n** as follows (for fermions *N*/*M* ≤ *δ* < 1)

21 $${Q}^{(B,F)}=\frac{\displaystyle \prod _{i=1}^{r}{(1\pm {n}_{i}/{K}_{i})}^{{n}_{i}\pm {K}_{i}-1/2}}{{(1\pm N/M)}^{N\pm M-1/2}}[1+O(\frac{r}{{\rm{\min }}({K}_{i})})].$$

Using simple algebraic manipulations one can expose in the logarithm of the right-hand-side of Eq. (21) a Kullback-Leibler divergence multiplied by a large negative factor, similar as in Eq. (15). Setting *α* = *N*/*M*, introducing ${X}_{i}\equiv \frac{{q}_{i}\pm \alpha {x}_{i}}{1\pm \alpha }$, and observing that 0 ≤ *X*_*i*_ ≤ 1 and $\displaystyle {\sum }_{i=1}^{r}{X}_{i}=1$ (an analog of *x*_*i*_ in Eqs (15)–(16)) we have

22 $$\mathrm{ln}\,{Q}^{(B,F)}=-\frac{r-1}{2}\,\mathrm{ln}(1\pm \alpha )-\frac{1}{2}\sum _{i=1}^{r}\,\mathrm{ln}({X}_{i}/{q}_{i})\pm M(1\pm \alpha )\sum _{i=1}^{r}{X}_{i}\,\mathrm{ln}({X}_{i}/{q}_{i})+O(\frac{r}{{\rm{\min }}({K}_{i})}).$$

Expanding the Kullback-Leibler divergence (the third term) on the right-hand-side of Eq. (22) as in Eq. (16), using that ${X}_{i}-{q}_{i}=\pm \frac{\alpha }{1\pm \alpha }({x}_{i}-{q}_{i})$ and replacing *X*_*i*_ by *q*_*i*_ in the second term (for similar reasons as in the classical case) one arrives at the following Gaussian approximation for the quantum factor

23 $${Q}^{(B,F)}\approx \frac{\exp \{\pm \frac{N\alpha }{1\pm \alpha }\displaystyle \sum _{i=1}^{r}\frac{{({x}_{i}-{q}_{i})}^{2}}{2{q}_{i}}\}}{{(1\pm \alpha )}^{\frac{r-1}{2}}}.$$

Multiplying the two factors in Eqs (17) and (23) we arrive at the asymptotic Gaussian of Eq. (5).

### The counting probability in the output bins of a given multiport

Let us recall that the probability to detect *N* identical particles in the output ports **l** = (*l*_1_, …, *l*_*N*_), corresponding to occupations **m** = (*m*_1_, …, *m*_*M*_), for an input **k** = (*k*_1_, …, *k*_*N*_), corresponding to occupations **s** = (*s*_1_, …, *s*_*M*_), reads^32, 34^

24 $$p({\bf{l}}|{\bf{k}})=\hat{p}({\bf{m}}|{\bf{s}})=\frac{1}{{\bf{m}}!{\bf{s}}!}\sum _{\tau ,\sigma \in {{\cal{S}}}_{N}}J(\tau {\sigma }^{-1})\prod _{a=1}^{N}{U}_{{k}_{\tau (a)},{l}_{a}}^{\ast }{U}_{{k}_{\sigma (a)},{l}_{a}},$$

where a unitary matrix *U* expands the input ports of a multiport over the output ones $\left|{k}^{(in)}\right\rangle =\displaystyle {\sum }_{l=1}^{M}{U}_{kl}\left|{l}^{(out)}\right\rangle$ and ${\bf{m}}!\equiv {\prod }_{l=1}^{M}{m}_{l}!$. In Eq. (24) the function *J*(*σ*) of the permutation *σ* of *N* objects, describing the state of partial distinguishability of the particles, is given as follows

25 $$J(\sigma )=\varepsilon (\sigma ){\rm{Tr}}({\rho }^{(\mathrm{int})}{P}_{\sigma }),\quad \varepsilon (\sigma )=\left\{\begin{array}{cc}1, & \mathrm{Bosons},\\ {\rm{sgn}}(\sigma ), & \mathrm{Fermions},\end{array}\right.$$

where ${P}_{\sigma }{\prod }_{a=1}^{N}\otimes \left|{j}_{a}\right\rangle ={\prod }_{a=1}^{N}\otimes \left|{j}_{{\sigma }^{-1}(a)}\right\rangle$ is an operator representation of *σ* in the Hilbert space of the internal states of *N* particles and *ρ* ^(*int*)^ is their internal state^32, 34^ (more details in Supplementary Information). Consider now a partition of the output ports of a *M*-port unitary network *U* into *r* bins with **K** = (*K*_1_, …, *K*_*r*_) ports. Summing up the probabilities of Eq. (24) with the occupations **m** = (**m** ^(1)^, …, **m** ^(*r*)^) corresponding to counting of **n** = (*n*_1_, …, *n*_*r*_) particles in *r* output bins gives

26 $$\begin{array}{rcl}P({\bf{n}};U) & = & \sum _{{\bf{m}}}^{\prime} \hat{p}({\bf{m}}|{\bf{s}})=\sum _{{\bf{l}}}^{\prime} \prod _{i=1}^{r}\frac{{{\bf{m}}}^{(i)}!}{{n}_{i}!}\hat{p}({\bf{m}}|{\bf{s}})\\  & = & \frac{1}{{\bf{s}}!{\bf{n}}!}\sum _{\sigma ,\tau \in {{\cal{S}}}_{N}}J(\sigma )\prod _{i=1}^{r}\sum _{{\bf{l}}}^{\prime} \prod _{a=1}^{N}{U}_{{k}_{\tau (a)},{l}_{a}}^{\ast }{U}_{{k}_{{\sigma }^{-1}\tau (a)},{l}_{a}}\\  & = & \frac{1}{{\bf{s}}!}\sum _{\sigma \in {{\cal{S}}}_{N}}J(\sigma )\sum _{{{\bf{a}}}_{1},\ldots ,{{\bf{a}}}_{r}}\prod _{i=1}^{r}\prod _{a\in {{\bf{a}}}_{i}}{H}_{a,\sigma (a)}^{(i)},\end{array}$$

where we have introduced for output bin *i* a *N*-dimensional positive semi-definite Hermitian matrix

27 $${H}_{a,b}^{(i)}\equiv \sum _{l\in {K}_{i}}{U}_{{k}_{a},l}{U}_{{k}_{b},l}^{\ast }$$

and a partition **a**_1_, …, **a**_*r*_ of the indices (1, …, *N*) into *r* subsets, such that vector **a**_*i*_ contains *n*_*i*_ indices. The partition represents the permutations of port indices complementary to the group *G*_**s**_ of symmetries of the input configuration **s** (with **s**! elements) in the group ${{\cal{S}}}_{N}$ of permutations of *N* objects. In the case of distinguishable particles we have^34^

28 $${J}^{(D)}(\sigma )=\sum _{\pi \in {G}_{{\bf{s}}}}{\delta }_{\sigma ,\pi }$$

which allows to further simplify the expression in Eq. (26) by computing the sum over *σ*. We get the factor **s**! (which cancels the same factor in the denominator) resulting in

29 $${P}^{(D)}({\bf{n}};U)=\sum _{{{\bf{a}}}_{1},\ldots ,{{\bf{a}}}_{r}}\prod _{i=1}^{r}\prod _{a\in {{\bf{a}}}_{i}}{H}_{a,\sigma (a)}^{(i)}\mathrm{.}$$

For bosons, in computation of the probability by Eq. (26) one cannot avoid an exponential in *N* number of the floating point operations. The numerical computation can be carried out using Ryser-Glynn’s formula for the matrix permanent^47^, by first computing the sum over *σ* for a fixed partition **a** (i.e., a product of the matrix permanents^41^) and then the sum over all possible partitions.

### Averaging the probability over the input configurations allowed by the statistics

Unitarity of the multiport matrix *U* results in equivalence of the averaging the probability *p*(**l**|**k**; *U*) of the transition **k** → **l** over the Haar-random unitary *U* to the averaging over all the input configurations **k**, allowed by the statistics, for an arbitrary (fixed) unitary *U*. Indeed, denoting the respective occupations by **s** and **m**, from Eq. (24) we have (see also ref. 48)

30 $${p}^{(F)}({\bf{l}}|{\bf{k}};U)={|{\rm{\det }}(U({\bf{k}}|{\bf{l}}))|}^{2},{p}^{(B)}({\bf{l}}|{\bf{k}};U)={\hat{p}}^{(B)}({\bf{m}}|{\bf{s}};U)=\frac{{|{\rm{per}}(U[{\bf{s}}|{\bf{m}}])|}^{2}}{{\bf{m}}!{\bf{s}}!}$$

where *U*(**k**|**l**) is a *N*-dimensional submatrix of *U* on the rows **k** and columns **l**, per(…) stands for the matrix permanent^41^ and the *N*-dimensional matrix *U*[**s**|**m**] is built by selecting rows and columns of *U* with the repetitions **s** and **m**, respectively. In both cases, the invariance properties of the matrix determinant and permanent imply the following time-inversion symmetry *p* ^(*B*,*F*)^(**l**|**k**; *U*) = *p* ^(*B*,*F*)^(**k**|**l**; *U* ^†^). On the other hand due to unitarity we must have

31 $$\sum _{|{\bf{m}}|=N}{\hat{p}}^{(B)}({\bf{m}}|{\bf{s}};U)=\mathrm{1,}\,\sum _{{\bf{l}}}{p}^{(F)}({\bf{l}}|{\bf{k}};U)=\mathrm{1,}$$

where in the case of bosons we sum over all **m** satisfying $|{\bf{m}}|\equiv {\sum }_{l=1}^{M}{m}_{l}=N$ and in the case of fermions over the sets of distinct indices **l** = (*l*_1_, …, *l*_*N*_). Using the time-invariance symmetry together with Eq. (31) in the definition of the average over all the (allowed) input configurations, we conclude, for instance, that

32 $${\left\langle {\hat{p}}^{(B)}({\bf{m}}\left|{\bf{s}};U\right.)\right\rangle }_{{\bf{s}}}=\frac{\displaystyle \sum _{|{\bf{s}}|=N}{\hat{p}}^{(B)}({\bf{s}}\left|{\bf{m}};{U}^{\dagger }\right.)}{\displaystyle \sum _{|{\bf{s}}|=N}1}={(\displaystyle \sum _{|{\bf{s}}|=N}1)}^{-1}=\frac{(M-\mathrm{1})!N!}{(M+N-\mathrm{1})!},$$

where we have counted the number of Fock states of *N* bosons in *M* ports. Since ${\left\langle {\hat{p}}^{(B)}({\bf{m}}\left|{\bf{s}};U\right.)\right\rangle }_{U}=\frac{(M-1)!N!}{(M+N-1)!}$ (see Supplementary Information) we obtain the announced result. Similar for fermions.

### The asymptotic average counting probability for a singular binning

Let us prove the asymptotic results in Eq. (14). For *N* → ∞ and *N*/*M* → *α* we obtain (by expanding the logarithm of the left-hand side)

33 $${(\frac{K}{M})}^{N}={(1-\frac{m}{M})}^{N}=\exp \{-N\sum _{p=1}^{\infty }\frac{1}{p}{(\frac{m}{M})}^{p}\}={e}^{-m\alpha }[1+O(\frac{1}{M})]\mathrm{.}$$

On the other hand, by applying Eq. (19) to the product in the quantum average in Eq. (13) we get

34 $$\begin{array}{rcl}\prod _{l=1}^{N-1}(\frac{1\pm l/K}{1\pm l/M}) & = & \frac{{(1\pm \frac{N-1}{K})}^{N\pm K-1/2}}{{(1\pm \frac{N-1}{M})}^{N\pm M-1/2}}[1+O(\frac{1}{M})]\\  & = & {(\frac{1\pm \frac{N-1}{K}}{1\pm \frac{N-1}{M}})}^{N\pm K-1/2}{(1\pm \frac{N-1}{M})}^{\mp m}[1+O(\frac{1}{M})]\\  & = & {(1\pm \frac{m(N-1)}{K(M\pm [N-1])})}^{N\pm K-1/2}{(1\pm \alpha )}^{\mp m}[1+O(\frac{1}{M})].\end{array}$$

The first factor on the right-hand side of Eq. (34) can be estimated similar as in Eq. (33)

35 $$\begin{array}{rcl}{(1\pm \frac{m(N-\mathrm{1})}{K(M\pm [N-\mathrm{1}])})}^{N\pm K-\mathrm{1/2}} & = & \exp \{(N\pm K-\mathrm{1/2})[\pm \frac{m(N-\mathrm{1})}{K(M\pm [N-\mathrm{1}])}+O(\frac{1}{{M}^{2}})]\}\\  & = & {e}^{m\alpha }[1+O(\frac{1}{M})]\mathrm{.}\end{array}$$

Using Eqs (33)–(35) into Eq. (13) we arrive at the asymptotic result stated in Eq. (14).

## Electronic Supplementary material

Supplementary Information
