# Supplementary material for: Asymptotic Gaussian law for noninteracting indistinguishable particles in random networks
Source: Sci Rep. 2017 Feb 16;7:31. doi: 10.1038/s41598-017-00044-8 (PMC5428393; doi:10.1038/s41598-017-00044-8)
Supplement: Supplementary file 1 — Supplementary Information [file 41598_2017_44_MOESM1_ESM.pdf]

Supplement to “Asymptotic Gaussian law for noninteracting indistinguishable particles in random networks”

V. S. Shchesnovich

Centro de Ciências Naturais e Humanas, Universidade Federal do ABC, Santo André, SP, 09210-170 Brazil

0.1 Probability of an output configuration of identical particles in a unitary linear multiport

We consider bosons and fermions simultaneously. The general state for N particles at the input of a M-port with occupations $\mathbf{s} = (s_1,\ldots,s_M)$ reads

$$\rho(\mathbf{s}) = \sum_i p_i |\Psi_i\rangle\langle\Psi_i|, \quad |\Psi_i\rangle = \frac{1}{\sqrt{\mathbf{s}!}} \sum_{\mathbf{j}} C^{(i)}_{\mathbf{j}} \prod_{a=1}^{N} \hat{a}^\dagger_{k_a,j_a}|0\rangle, \tag{1}$$

where $\hat{a}^\dagger_{k,j}$ is the creation operator in port k_a and a basis state $|j_a\rangle \in \mathscr{H}$ in the internal space, $\mathbf{j} = (j_1,\ldots,j_N)$, $p_i \geq 0$, $\sum_i p_i = 1$, and $\mathbf{s}! \equiv s_1!\cdot\ldots\cdot s_M!$. The permutation symmetry (anti-symmetry) of the operators for bosons (fermions) allows one to chose the expansion coefficients $C^{(i)}_{\mathbf{j}}$ to be symmetric (anti-symmetric) with respect to the symmetry subgroup $G_{\mathbf{s}} \equiv \mathscr{S}_{s_1}\otimes\ldots\otimes\mathscr{S}_{s_M}$ of the symmetric group $\mathscr{S}_N$, where $\mathscr{S}_{s_j}$ corresponds to the permutations of the internal states of the particles in the input port j between themselves. The symmetric (anti-symmetric) coefficients are normalized by $\sum_{\mathbf{j}} |C^{(i)}_{\mathbf{j}}|^2 = 1$. The probability to detect $\mathbf{m} = (m_1,\ldots,m_M)$ particles in the output ports with corresponding to the annihilation perators $\hat{b}_{l,j}$ reads[1,2]

$$p(\mathbf{m}|\mathbf{s}) = \mathrm{Tr}(\rho(\mathbf{s})\mathscr{D}(\mathbf{m})), \tag{2}$$

where ρ is the input state Eq. (1) and $\mathscr{D}(\mathbf{m})$ is the detection operator ($|0\rangle$ is the vacuum state)

$$\mathscr{D}(\mathbf{m}) = \frac{1}{\mathbf{m}!}\sum_{\mathbf{j}} \left[\prod_{a=1}^{N}\hat{b}^\dagger_{l_a,j_a}\right]|0\rangle\langle 0|\left[\prod_{a=1}^{N}\hat{b}_{l_a,j_a}\right]. \tag{3}$$

With $\varepsilon(\sigma) = 1$ for bosons and $\varepsilon(\sigma) = \mathrm{sgn}(\sigma)$ fermions, the following identity can be shown[1]

$$\langle 0|\left[\prod_{a=1}^{N}\hat{b}_{l_a,j_a}\right]\left[\prod_{a=1}^{N}\hat{b}^\dagger_{l'_a,j'_a}\right]|0\rangle = \sum_{\sigma\in\mathscr{S}_N}\varepsilon(\sigma)\prod_{a=1}^{N}\delta_{l'_a,l_{\sigma(a)}}\delta_{j'_a,j_{\sigma(a)}}, \tag{4}$$

where the summation is over the symmetric group $\mathscr{S}_N$ of permutations of N objects. Substituting Eqs. (2) and (3) into Eq. (1), introducing a unitary linear network U, $\hat{a}^\dagger_{k,j} = \sum_{l=1}^{M} U_{kl}\hat{b}^\dagger_{l,j}$, and using Eq. (4) one obtains the probability of an output configuration $\mathbf{m}$ in the form[1]

$$p(\mathbf{m}|\mathbf{s}) = \frac{1}{\mathbf{m}!\mathbf{s}!}\sum_{\tau,\sigma\in\mathscr{S}_N} J(\tau\sigma^{-1})\prod_{a=1}^{N} U^*_{k_{\tau(a)},l_a}U_{k_{\sigma(a)},l_a}, \tag{5}$$

where $l_1,\ldots,l_N$ are the output ports corresponding to occupations $(m_1,\ldots,m_M)$, whereas the function $J(\sigma)$ is defined as

$$J(\sigma) = \varepsilon(\sigma)\mathrm{Tr}\big(\rho^{(int)}P_\sigma\big), \quad \rho^{(int)} = \sum_i p_i|\psi_i\rangle\langle\psi_i|, \quad |\psi_i\rangle \equiv \sum_{\mathbf{j}} C^{(i)}_{\mathbf{j}} \prod_{a=1}^{N}{}^{\otimes}|j_a\rangle, \tag{6}$$

where $P_\sigma \prod_{a=1}^{N}{}^{\otimes}|j_a\rangle = \prod_{a=1}^{N}{}^{\otimes}|j_{\sigma^{-1}(a)}\rangle$ is the operator representation of σ in $\mathscr{H}^{\otimes N}$. Note that the permutation symmetry (anti-symmetry) of the input state (1) for bosons (fermions), i.e., $P_\pi\rho^{(int)} = \rho^{(int)}P_\pi = \varepsilon(\pi)\rho^{(int)}$ for any $\pi\in G_{\mathbf{s}}$, implies that[1]

$$J(\sigma\pi) = J(\pi\sigma) = J(\sigma), \quad \forall\pi\in G_{\mathbf{s}}. \tag{7}$$

Identical particles are called completely indistinguishable if $\rho^{(int)}$ is symmetric under permutations, $J^{(id)}(\sigma) = \varepsilon(\sigma)$ (e.g., identical particles in the same internal state), whereas in the case of distinguishable particles we have[1,2]

$$J^{(D)}(\sigma) = \sum_{\pi\in G_{\mathbf{s}}}\delta_{\sigma,\pi}. \tag{8}$$

Finally, one can easily establish that in the case of completely indistinguishable bosons the probability of an output configuration $\mathbf{m}$ for an input $\mathbf{s}$ is[3]

$$p^{(B)}(\mathbf{m}|\mathbf{s};U) = \frac{|\mathrm{per}(U[\mathbf{s}|\mathbf{m}])|^2}{\mathbf{s}!\mathbf{m}!}, \tag{9}$$

where $\mathrm{per}(\ldots)$ stands for the matrix permanent[4], the N-dimensional matrix $U[\mathbf{s}|\mathbf{m}]$ is built from a unitary matrix U by taking rows and columns with multiplicities $\mathbf{s}$ and $\mathbf{m}$, respectively. For completely indistinguishable fermions the probability of detecting particles in output ports $\mathbf{l} = (l_1,\ldots,l_N)$, $l_j \neq l_i$ for $i \neq j$, for an input $\mathbf{k}$ reads

$$p^{(F)}(\mathbf{l}|\mathbf{k};U) = |\det(U(\mathbf{k}|\mathbf{l}))|^2. \tag{10}$$

0.2 The average probability of the output configurations in a Haar-random unitary network

Let us show that the average probability in a Haar-random unitary network for bosons and fermions is uniform over the input and output configurations allowed by the quantum statistics. The average of a multinomial in U_{kl} and U^*_{mj} over the Haar-random M-dimensional unitary matrix U involves the Weingarten function $\mathscr{W}(M,\sigma)$[5,6] of permutations σ,

$$\langle \prod_{a=1}^{N} U_{k_a,l_a} U^*_{k'_a,l'_a}\rangle = \sum_{\nu,\tau\in\mathscr{S}_N} \mathscr{W}(M,\nu\tau^{-1}) \prod_{a=1}^{N} \delta_{k'_a,k_{\nu(a)}} \delta_{l'_a,l_{\tau(a)}}, \tag{11}$$

which depends only on the cycle structure of the permutation $\sigma = \nu\tau^{-1}$, i.e., the sequence of the numbers $(c_1(\sigma),\ldots,c_N(\sigma))$ of cycles of lengths $(1,\ldots,N)$ in the cycle decomposition of $\sigma \in \mathscr{S}_N$[8]. Using Eq. (11) in the averaging of the probability in Eq. (5) we have

$$\begin{aligned}\langle p(\mathbf{m}|\mathbf{s})\rangle_U &= \frac{1}{\mathbf{m}!\mathbf{s}!} \sum_{\sigma,\tau\in\mathscr{S}_N} J(\tau\sigma^{-1}) \langle \prod_{k=1}^{N} U_{k_{\sigma(a)},l_a} U^*_{k_{\tau(a)},l_a}\rangle = \frac{1}{\mathbf{m}!\mathbf{s}!} \sum_{\sigma,\tau\in\mathscr{S}_N} J(\tau\sigma^{-1}) \sum_{\nu\in G_{\mathbf{s}},\pi\in G_{\mathbf{m}}} \mathscr{W}(M,\tau^{-1}\sigma\nu\pi) \\ &= \frac{N!}{\mathbf{m}!} \sum_{\sigma\in\mathscr{S}_N} J(\sigma) \sum_{\pi\in G_{\mathbf{m}}} \mathscr{W}(M,\sigma^{-1}\pi) = \frac{N!}{\mathbf{m}!} \sum_{\sigma\in\mathscr{S}_N} J(\sigma) \sum_{\pi\in G_{\mathbf{m}}} \mathscr{W}(M,\sigma\pi)\end{aligned} \tag{12}$$

where the symmetry of $J(\sigma)$ in Eq. (7) and the dependence of $\mathscr{W}(\sigma)$ only on the cycle structure of σ were used.

For indistinguishable bosons (fermions) $J^{(B)}(\sigma) = 1$ ($J^{(F)}(\sigma) = \mathrm{sgn}(\sigma)$). In this case, the substitution $\sigma \to \sigma\pi$ induces factoring of the two summations in Eq. (12), the sum over $\tau \in \mathscr{S}_{\mathbf{m}}$ cancels the denominator in Eq. (12) and the identities

$$\sum_{\sigma\in\mathscr{S}_N} \mathscr{W}(M,\sigma) = \frac{(M-1)!}{(M+N-1)!}, \quad \sum_{\sigma\in\mathscr{S}_N} \mathrm{sgn}(\sigma)\mathscr{W}(M,\sigma) = \frac{(M-N)!}{M!} \tag{13}$$

follow from the fact that the probabilities must sum to 1. From these expressions and the fact that $G_{\mathbf{m}} \subset \mathscr{S}_N$ the result for indistinguishable bosons or fermions easily follows (in case of bosons for arbitrary input).

For distinguishable particles an approximate expression can be easily derived for the single particles at the input, $s_j \leq 1$ (distinguishable particles from the same input port are not distinguished by a linear multiport from the indistinguishable bosons, hence the expression in the general case is complicated). In this case $J^{(D)}(\sigma) = \delta_{\sigma,I}$, where I is the identity permutation, we obtain

$$\langle p^{(D)}(\mathbf{m}|\mathbf{s})\rangle_U = \frac{N!}{\mathbf{m}!} \sum_{\pi\in G_{\mathbf{m}}} \mathscr{W}(M,\pi). \tag{14}$$

First of all, Eq. (14) gives the exact average for N distinguishable particles in a Haar-random network and does not coincide with Eq. (1) of the main text (valid for the averaging for the particle propagating one at a time in a new random network). Indeed, for an output configuration with up to one particle per port we get from Eq. (14) $\langle p^{(D)}(\mathbf{m}|\mathbf{s})\rangle_U = N!\mathscr{W}(M,I_N) \neq N!/M^N$ (with I_N being the trivial permutation) as can be verified using some known examples[7]. Nevertheless, the two expressions are close for $M \gg 1$.

An approximate evaluation of the expression in Eq. (14) is possible by observing that $\pi = \pi_1 \otimes \ldots \otimes \pi_M$, where $\pi \in \mathscr{S}_{m_l}$, and employing the following asymptotic formula for $\sigma \in S_m$[6]

$$\mathscr{W}(M,\sigma) \approx \frac{(-1)^m}{M^{2m}} \prod_{i=1}^{m} (-Mg_i)^{c_i(\sigma)}, \quad g_i \equiv \frac{(2i-2)!}{i!(i-1)!}. \tag{15}$$

Eq. (15) ensures an approximate factorization $\mathscr{W}(M,\pi) \approx \mathscr{W}(M,\pi_1)\ldots\mathscr{W}(M,\pi_M)$ by the additivity of the number of cycles of length i[8] $c_i(\pi_1 \otimes \ldots \otimes \pi_M) = c_i(\pi_1) + \ldots c_i(\pi_M)$. Therefore, the r.h.s. of Eq. (14) will depend on the product of the following summations

$$Z_m \equiv \sum_{\pi \in \mathscr{S}_m} \prod_{i=1}^{m} (-Mg_i)^{c_i(\pi)} = \frac{d^m F(x)}{dx^m}\Big|_{x=0}, \tag{16}$$

where $F(x)$ is the generating function[8], which in our case reads (see the Supplement to Ref.[2])

$$F(x) = \left(\frac{2}{\sqrt{1-4x}+1} \exp\left\{ \sqrt{1-4x} - 1 \right\} \right)^M. \tag{17}$$

We have $dF(x)/dx = MF(x)/\Phi(x)$ where $\Phi(x) = -(1+\sqrt{1-4x})/2$. One can prove by induction that

$$\frac{d^m F(x)}{dx^m} = \left(\frac{M}{\Phi(x)} \right)^m F(x) \left[1 - \frac{m(m-1)}{2M} + O\left(M^{-2}\right) \right]. \tag{18}$$

By Eqs. (16) and (18) $Z_m = (-M)^m[1 - m(m-1)/2M + O(M^{-2})]$ and we obtain from Eqs. (14) and (15)

$$\langle p^{(D)}(\mathbf{m}|\mathbf{s})\rangle_U \approx \frac{N!}{M^N \mathbf{m}!} \left[1 - \sum_{l=1}^{M} \frac{m_l(m_l-1)}{2M} \right]. \tag{19}$$

The error in Eq. (19) can be easily estimated by observing that the first factor gives the normalized probabilities which sum to 1, whereas we get for the second term

$$\sum_{\mathbf{m}} \frac{N!}{M^N \mathbf{m}!} \sum_{l=1}^{M} \frac{m_l(m_l-1)}{2M} = \frac{1}{2M^{N+1}} \sum_{l=1}^{M} \frac{\partial^2}{\partial x_l^2} \left(\sum_{l=1}^{M} x_l \right)^N \Bigg|_{x_l=1} = \frac{N(N-1)}{2M^2}. \tag{20}$$

Therefore, Eq. (19) is a good approximation for $(N/M)^2 \equiv \alpha^2 \ll 1$. Eq. (19) differs from the the expression in Eq. (1) of the main text (valid for sending one particle at a time through a Haar-random multiport U) by the factor in the square brackets accounting for the correlations between the transition matrix elements $A_{kl} \equiv |U_{k,l}|^2$ when N simultaneous distinguishable particles are at the input.

References

1. Shchesnovich, V. S. *Phys. Rev. A* **89**, 022333 (2014); *ibid* **91**, 013844 (2015).
2. Shchesnovich, V. S. *Phys. Rev. Lett.* **116**, 123601 (2016).
3. Scheel, S. arXiv:quant-ph/0406127.
4. Minc, H. *Permanents, Encyclopedia of Mathematics and Its Applications*, Vol. **6** (Addison-Wesley Publ. Co., Reading, Mass., 1978).
5. Weingarten, D. *J. Math. Phys.* **19**, 999 (1978).
6. Brouwer, P. W. & Beenakker, C. W. J. *J. Math. Phys.* **37**, 4904 (1996).
7. Novak, J. arXiv:0811.3595 [math.CO].
8. Stanley, R. P. *Enumerative Combinatorics*, 2nd ed., Vol. 1 (Cambridge University Press, 2011).
